# Supplementary material for: Resolving the full spectrum of human genome variation using Linked-Reads
Source: Genome Res. 2019 Apr;29(4):635–45. doi: 10.1101/gr.234443.118 (PMC6442396; doi:10.1101/gr.234443.118)
Supplement: Supplemental Material [file supp_gr.234443.118_Supplemental_longranger-2.2.zip › longranger-2.2.tar.bz2!/lr2.2-public/lib/stan/cmdstan/stan/doc/api/html/bfgs_8hpp_source.html]

Stan: src/stan/optimization/bfgs.hpp Source File


|  |  |
| --- | --- |
|  | Stan  2.5.0  probability, sampling & optimization |


- Main Page
- Related Pages
- Namespaces
- Classes
- Files

- File List
- File Members

All Classes Namespaces Files Functions Variables Typedefs Enumerations Enumerator Friends Macros Pages

- src
- stan
- optimization

bfgs.hpp

Go to the documentation of this file.

1 #ifndef STAN\_\_OPTIMIZATION\_\_BFGS\_HPP

2 #define STAN\_\_OPTIMIZATION\_\_BFGS\_HPP

3

4 #include <cmath>

5 #include <cstdlib>

6 #include <string>

7 #include <limits>

8

9 #include <boost/math/special\_functions/fpclassify.hpp>

10

11 #include <stan/math/matrix/Eigen.hpp>

12 #include <stan/math/matrix/meta/index\_type.hpp>

13 #include <stan/model/util.hpp>

14

15 #include <stan/optimization/bfgs\_linesearch.hpp>

16 #include <stan/optimization/bfgs\_update.hpp>

17 #include <stan/optimization/lbfgs\_update.hpp>

18

19 namespace stan {

20  namespace optimization {

21  typedef enum {

22  TERM\_SUCCESS = 0,

23  TERM\_ABSX = 10,

24  TERM\_ABSF = 20,

25  TERM\_RELF = 21,

26  TERM\_ABSGRAD = 30,

27  TERM\_RELGRAD = 31,

28  TERM\_MAXIT = 40,

29  TERM\_LSFAIL = -1

30  } TerminationCondition;

31

32  template<typename Scalar = double>

33  class ConvergenceOptions {

34  public:

35  ConvergenceOptions() {

36  maxIts = 10000;

37  fScale = 1.0;

38

39  tolAbsX = 1e-8;

40  tolAbsF = 1e-12;

41  tolAbsGrad = 1e-8;

42

43  tolRelF = 1e+4;

44  tolRelGrad = 1e+3;

45  }

46  size\_t maxIts;

47  Scalar tolAbsX;

48  Scalar tolAbsF;

49  Scalar tolRelF;

50  Scalar fScale;

51  Scalar tolAbsGrad;

52  Scalar tolRelGrad;

53  };

54

55  template<typename Scalar = double>

56  class LSOptions {

57  public:

58  LSOptions() {

59  c1 = 1e-4;

60  c2 = 0.9;

61  minAlpha = 1e-12;

62  alpha0 = 1e-3;

63  }

64  Scalar c1;

65  Scalar c2;

66  Scalar alpha0;

67  Scalar minAlpha;

68  };

69

70

71  template<typename FunctorType, typename QNUpdateType,

72  typename Scalar = double, int DimAtCompile = Eigen::Dynamic>

73  class BFGSMinimizer {

74  public:

75  typedef Eigen::Matrix<Scalar,DimAtCompile,1> VectorT;

76  typedef Eigen::Matrix<Scalar,DimAtCompile,DimAtCompile> HessianT;

77

78  protected:

79  FunctorType &\_func;

80  VectorT \_gk, \_gk\_1, \_xk\_1, \_xk, \_pk, \_pk\_1;

81  Scalar \_fk, \_fk\_1, \_alphak\_1;

82  Scalar \_alpha, \_alpha0;

83  size\_t \_itNum;

84  std::string \_note;

85  QNUpdateType \_qn;

86

87  public:

88  LSOptions<Scalar> \_ls\_opts;

89  ConvergenceOptions<Scalar> \_conv\_opts;

90

91  QNUpdateType &get\_qnupdate() { return \_qn; }

92  const QNUpdateType &get\_qnupdate() const { return \_qn; }

93

94  const Scalar &curr\_f() const { return \_fk; }

95  const VectorT &curr\_x() const { return \_xk; }

96  const VectorT &curr\_g() const { return \_gk; }

97  const VectorT &curr\_p() const { return \_pk; }

98

99  const Scalar &prev\_f() const { return \_fk\_1; }

100  const VectorT &prev\_x() const { return \_xk\_1; }

101  const VectorT &prev\_g() const { return \_gk\_1; }

102  const VectorT &prev\_p() const { return \_pk\_1; }

103  Scalar prev\_step\_size() const { return \_pk\_1.norm()\*\_alphak\_1; }

104

105  inline Scalar rel\_grad\_norm() const {

106  return -\_pk.dot(\_gk) / std::max(std::fabs(\_fk),\_conv\_opts.fScale);

107  }

108  inline Scalar rel\_obj\_decrease() const {

109  return std::fabs(\_fk\_1 - \_fk) / std::max(std::fabs(\_fk\_1),

110  std::max(std::fabs(\_fk),

111  \_conv\_opts.fScale));

112  }

113

114  const Scalar &alpha0() const { return \_alpha0; }

115  const Scalar &alpha() const { return \_alpha; }

116  const size\_t iter\_num() const { return \_itNum; }

117

118  const std::string &note() const { return \_note; }

119

120  std::string get\_code\_string(int retCode) {

121  switch(retCode) {

122  case TERM\_SUCCESS:

123  return std::string("Successful step completed");

124  case TERM\_ABSF:

125  return std::string("Convergence detected: absolute change in objective function was below tolerance");

126  case TERM\_RELF:

127  return std::string("Convergence detected: relative change in objective function was below tolerance");

128  case TERM\_ABSGRAD:

129  return std::string("Convergence detected: gradient norm is below tolerance");

130  case TERM\_RELGRAD:

131  return std::string("Convergence detected: relative gradient magnitude is below tolerance");

132  case TERM\_ABSX:

133  return std::string("Convergence detected: absolute parameter change was below tolerance");

134  case TERM\_MAXIT:

135  return std::string("Maximum number of iterations hit, may not be at an optima");

136  case TERM\_LSFAIL:

137  return std::string("Line search failed to achieve a sufficient decrease, no more progress can be made");

138  default:

139  return std::string("Unknown termination code");

140  }

141  }

142

143  BFGSMinimizer(FunctorType &f) : \_func(f) { }

144

145  void initialize(const VectorT &x0) {

146  int ret;

147  \_xk = x0;

148  ret = \_func(\_xk,\_fk,\_gk);

149  if (ret) {

150  throw std::runtime\_error("Error evaluating initial BFGS point.");

151  }

152  \_pk = -\_gk;

153

154  \_itNum = 0;

155  \_note = "";

156  }

157

158  int step() {

159  Scalar gradNorm, stepNorm;

160  VectorT sk, yk;

161  int retCode(0);

162  int resetB(0);

163

164  \_itNum++;

165

166  if (\_itNum == 1) {

167  resetB = 1;

168  \_note = "";

169  }

170  else {

171  resetB = 0;

172  \_note = "";

173  }

174

175  while (true) {

176  if (resetB) {

177  // Reset the Hessian approximation

178  \_pk.noalias() = -\_gk;

179  }

180

181  // Get an initial guess for the step size (alpha)

182  if (\_itNum > 1 && resetB != 2) {

183  // use cubic interpolation based on the previous step

184  \_alpha0 = \_alpha = std::min(1.0,

185  1.01\*CubicInterp(\_gk\_1.dot(\_pk\_1),

186  \_alphak\_1,

187  \_fk - \_fk\_1,

188  \_gk.dot(\_pk\_1),

189  \_ls\_opts.minAlpha, 1.0));

190  }

191  else {

192  // On the first step (or, after a reset) use the default step size

193  \_alpha0 = \_alpha = \_ls\_opts.alpha0;

194  }

195

196  // Perform the line search. If successful, the results are in the

197  // variables: \_xk\_1, \_fk\_1 and \_gk\_1.

198  retCode = WolfeLineSearch(\_func, \_alpha, \_xk\_1, \_fk\_1, \_gk\_1,

199  \_pk, \_xk, \_fk, \_gk,

200  \_ls\_opts.c1, \_ls\_opts.c2,

201  \_ls\_opts.minAlpha);

202  if (retCode) {

203  // Line search failed...

204  if (resetB) {

205  // did a Hessian reset and it still failed, and nothing left to try

206  retCode = TERM\_LSFAIL;

207  return retCode;

208  }

209  else {

210  // try resetting the Hessian approximation

211  resetB = 2;

212  \_note += "LS failed, Hessian reset";

213  continue;

214  }

215  }

216  else {

217  break;

218  }

219  }

220

221  // Swap things so that k is the most recent iterate

222  std::swap(\_fk,\_fk\_1);

223  \_xk.swap(\_xk\_1);

224  \_gk.swap(\_gk\_1);

225  \_pk.swap(\_pk\_1);

226

227  sk.noalias() = \_xk - \_xk\_1;

228  yk.noalias() = \_gk - \_gk\_1;

229

230  gradNorm = \_gk.norm();

231  stepNorm = sk.norm();

232

233  // Update QN approximation

234  if (resetB) {

235  // If the QN approximation was reset, automatically scale it

236  // and update the step-size accordingly

237  Scalar B0fact = \_qn.update(yk,sk,true);

238  \_pk\_1 /= B0fact;

239  \_alphak\_1 = \_alpha\*B0fact;

240  }

241  else {

242  \_qn.update(yk,sk);

243  \_alphak\_1 = \_alpha;

244  }

245

246

247  // Compute search direction for next step

248  \_qn.search\_direction(\_pk,\_gk);

249

250  // Check for convergence

251  if (std::fabs(\_fk\_1 - \_fk) < \_conv\_opts.tolAbsF) {

252  retCode = TERM\_ABSF; // Objective function improvement wasn't sufficient

253  }

254  else if (gradNorm < \_conv\_opts.tolAbsGrad) {

255  retCode = TERM\_ABSGRAD; // Gradient norm was below threshold

256  }

257  else if (stepNorm < \_conv\_opts.tolAbsX) {

258  retCode = TERM\_ABSX; // Change in x was too small

259  }

260  else if (\_itNum >= \_conv\_opts.maxIts) {

261  retCode = TERM\_MAXIT; // Max number of iterations hit

262  }

263  else if (rel\_obj\_decrease() < \_conv\_opts.tolRelF\*std::numeric\_limits<Scalar>::epsilon()) {

264  retCode = TERM\_RELF; // Relative improvement in objective function wasn't sufficient

265  }

266  else if (rel\_grad\_norm() < \_conv\_opts.tolRelGrad\*std::numeric\_limits<Scalar>::epsilon()) {

267  retCode = TERM\_RELGRAD; // Relative gradient norm was below threshold

268  }

269  else {

270  retCode = TERM\_SUCCESS; // Step was successful more progress to be made

271  }

272

273  return retCode;

274  }

275

276  int minimize(VectorT &x0) {

277  int retcode;

278  initialize(x0);

279  while (!(retcode = step()));

280  x0 = \_xk;

281  return retcode;

282  }

283  };

284

285

286

287  template <typename M>

288  double lp\_no\_jacobian(const M& model,

289  std::vector<double>& params\_r,

290  std::vector<int>& params\_i,

291  std::ostream\* o = 0) {

292  // FIXME: is this supposed to return the log probability from the model?

293  return 0;

294  }

295

296  template <class M>

297  class ModelAdaptor {

298  private:

299  M& \_model;

300  std::vector<int> \_params\_i;

301  std::ostream\* \_msgs;

302  std::vector<double> \_x, \_g;

303  size\_t \_fevals;

304

305  public:

306  ModelAdaptor(M& model,

307  const std::vector<int>& params\_i,

308  std::ostream\* msgs)

309  : \_model(model), \_params\_i(params\_i), \_msgs(msgs), \_fevals(0) {}

310

311  size\_t fevals() const { return \_fevals; }

312  int operator()(const Eigen::Matrix<double,Eigen::Dynamic,1> &x,

313  double &f) {

314  using Eigen::Matrix;

315  using Eigen::Dynamic;

316  using stan::math::index\_type;

317  using stan::model::log\_prob\_propto;

318  typedef typename index\_type<Matrix<double,Dynamic,1> >::type idx\_t;

319

320  \_x.resize(x.size());

321  for (idx\_t i = 0; i < x.size(); i++)

322  \_x[i] = x[i];

323

324  try {

325  f = - log\_prob\_propto<false>(\_model, \_x, \_params\_i, \_msgs);

326  } catch (const std::exception& e) {

327  if (\_msgs)

328  (\*\_msgs) << e.what() << std::endl;

329  return 1;

330  }

331

332  if (boost::math::isfinite(f))

333  return 0;

334  else {

335  if (\_msgs)

336  \*\_msgs << "Error evaluating model log probability: "

337  "Non-finite function evaluation." << std::endl;

338  return 2;

339  }

340  }

341  int operator()(const Eigen::Matrix<double,Eigen::Dynamic,1> &x,

342  double &f,

343  Eigen::Matrix<double,Eigen::Dynamic,1> &g) {

344  using Eigen::Matrix;

345  using Eigen::Dynamic;

346  using stan::math::index\_type;

347  using stan::model::log\_prob\_grad;

348  typedef typename index\_type<Matrix<double,Dynamic,1> >::type idx\_t;

349

350  \_x.resize(x.size());

351  for (idx\_t i = 0; i < x.size(); i++)

352  \_x[i] = x[i];

353

354  \_fevals++;

355

356  try {

357  f = - log\_prob\_grad<true,false>(\_model, \_x, \_params\_i, \_g, \_msgs);

358  } catch (const std::exception& e) {

359  if (\_msgs)

360  (\*\_msgs) << e.what() << std::endl;

361  return 1;

362  }

363

364  g.resize(\_g.size());

365  for (size\_t i = 0; i < \_g.size(); i++) {

366  if (!boost::math::isfinite(\_g[i])) {

367  if (\_msgs)

368  \*\_msgs << "Error evaluating model log probability: "

369  "Non-finite gradient." << std::endl;

370  return 3;

371  }

372  g[i] = -\_g[i];

373  }

374

375  if (boost::math::isfinite(f))

376  return 0;

377  else {

378  if (\_msgs)

379  \*\_msgs << "Error evaluating model log probability: "

380  "Non-finite function evaluation."

381  << std::endl;

382  return 2;

383  }

384  }

385  int df(const Eigen::Matrix<double,Eigen::Dynamic,1> &x,

386  Eigen::Matrix<double,Eigen::Dynamic,1> &g) {

387  double f;

388  return (\*this)(x,f,g);

389  }

390  };

391

392  template<typename M, typename QNUpdateType, typename Scalar = double,

393  int DimAtCompile = Eigen::Dynamic>

394  class BFGSLineSearch : public BFGSMinimizer<ModelAdaptor<M>,QNUpdateType,Scalar,DimAtCompile> {

395  private:

396  ModelAdaptor<M> \_adaptor;

397  public:

398  typedef BFGSMinimizer<ModelAdaptor<M>,QNUpdateType,Scalar,DimAtCompile> BFGSBase;

399  typedef typename BFGSBase::VectorT vector\_t;

400  typedef typename stan::math::index\_type<vector\_t>::type idx\_t;

401

402  BFGSLineSearch(M& model,

403  const std::vector<double>& params\_r,

404  const std::vector<int>& params\_i,

405  std::ostream\* msgs = 0)

406  : BFGSBase(\_adaptor),

407  \_adaptor(model,params\_i,msgs)

408  {

409  initialize(params\_r);

410  }

411

412  void initialize(const std::vector<double>& params\_r) {

413  Eigen::Matrix<double,Eigen::Dynamic,1> x;

414  x.resize(params\_r.size());

415  for (size\_t i = 0; i < params\_r.size(); i++)

416  x[i] = params\_r[i];

417  BFGSBase::initialize(x);

418  }

419

420  size\_t grad\_evals() { return \_adaptor.fevals(); }

421  double logp() { return -(this->curr\_f()); }

422  double grad\_norm() { return this->curr\_g().norm(); }

423  void grad(std::vector<double>& g) {

424  const vector\_t &cg(this->curr\_g());

425  g.resize(cg.size());

426  for (idx\_t i = 0; i < cg.size(); i++)

427  g[i] = -cg[i];

428  }

429  void params\_r(std::vector<double>& x) {

430  const vector\_t &cx(this->curr\_x());

431  x.resize(cx.size());

432  for (idx\_t i = 0; i < cx.size(); i++)

433  x[i] = cx[i];

434  }

435  };

436

437  }

438

439 }

440

441 #endif

stan::optimization::ConvergenceOptions::maxIts

size\_t maxIts

**Definition:** bfgs.hpp:46

stan::optimization::BFGSMinimizer::\_note

std::string \_note

**Definition:** bfgs.hpp:84

stan::optimization::BFGSMinimizer::iter\_num

const size\_t iter\_num() const

**Definition:** bfgs.hpp:116

stan::optimization::TERM\_MAXIT

**Definition:** bfgs.hpp:28

stan::optimization::BFGSMinimizer::\_alpha

Scalar \_alpha

**Definition:** bfgs.hpp:82

stan::agrad::fabs

fvar< T > fabs(const fvar< T > &x)

**Definition:** fabs.hpp:18

stan::optimization::TERM\_SUCCESS

**Definition:** bfgs.hpp:22

stan::optimization::BFGSLineSearch::params\_r

void params\_r(std::vector< double > &x)

**Definition:** bfgs.hpp:429

stan::optimization::BFGSMinimizer::get\_qnupdate

QNUpdateType & get\_qnupdate()

**Definition:** bfgs.hpp:91

stan::optimization::BFGSLineSearch::grad\_norm

double grad\_norm()

**Definition:** bfgs.hpp:422

stan::optimization::BFGSMinimizer::alpha0

const Scalar & alpha0() const

**Definition:** bfgs.hpp:114

stan::optimization::BFGSMinimizer::\_alphak\_1

Scalar \_alphak\_1

**Definition:** bfgs.hpp:81

stan::optimization::ModelAdaptor::ModelAdaptor

ModelAdaptor(M &model, const std::vector< int > &params\_i, std::ostream \*msgs)

**Definition:** bfgs.hpp:306

stan::model::log\_prob\_grad

double log\_prob\_grad(const M &model, std::vector< double > &params\_r, std::vector< int > &params\_i, std::vector< double > &gradient, std::ostream \*msgs=0)

Compute the gradient using reverse-mode automatic differentiation, writing the result into the specif...

**Definition:** util.hpp:83

stan::optimization::ModelAdaptor

**Definition:** bfgs.hpp:297

stan::optimization::BFGSMinimizer::curr\_x

const VectorT & curr\_x() const

**Definition:** bfgs.hpp:95

stan::optimization::BFGSMinimizer::\_conv\_opts

ConvergenceOptions< Scalar > \_conv\_opts

**Definition:** bfgs.hpp:89

stan::optimization::TerminationCondition

TerminationCondition

**Definition:** bfgs.hpp:21

stan::optimization::LSOptions::c1

Scalar c1

**Definition:** bfgs.hpp:64

stan::optimization::BFGSMinimizer::\_xk

VectorT \_xk

**Definition:** bfgs.hpp:80

stan::optimization::BFGSMinimizer::prev\_g

const VectorT & prev\_g() const

**Definition:** bfgs.hpp:101

stan::optimization::BFGSLineSearch::BFGSBase

BFGSMinimizer< ModelAdaptor< M >, QNUpdateType, Scalar, DimAtCompile > BFGSBase

**Definition:** bfgs.hpp:398

stan::optimization::TERM\_ABSX

**Definition:** bfgs.hpp:23

stan::optimization::BFGSMinimizer::BFGSMinimizer

BFGSMinimizer(FunctorType &f)

**Definition:** bfgs.hpp:143

boost::math::isfinite

bool isfinite(const stan::agrad::var &v)

Checks if the given number has finite value.

**Definition:** boost\_fpclassify.hpp:39

stan::optimization::BFGSMinimizer::\_pk\_1

VectorT \_pk\_1

**Definition:** bfgs.hpp:80

stan::optimization::ConvergenceOptions

**Definition:** bfgs.hpp:33

stan::optimization::WolfeLineSearch

int WolfeLineSearch(FunctorType &func, Scalar &alpha, XType &x1, Scalar &f1, XType &gradx1, const XType &p, const XType &x0, const Scalar &f0, const XType &gradx0, const Scalar &c1, const Scalar &c2, const Scalar &minAlpha)

Perform a line search which finds an approximate solution to: satisfying the strong Wolfe conditions...

**Definition:** bfgs\_linesearch.hpp:225

stan::optimization::BFGSLineSearch::idx\_t

stan::math::index\_type< vector\_t >::type idx\_t

**Definition:** bfgs.hpp:400

stan::optimization::ConvergenceOptions::tolAbsX

Scalar tolAbsX

**Definition:** bfgs.hpp:47

stan::math::max

double max(const double a, const double b)

**Definition:** max.hpp:7

stan::optimization::BFGSMinimizer::prev\_f

const Scalar & prev\_f() const

**Definition:** bfgs.hpp:99

stan::optimization::BFGSMinimizer::VectorT

Eigen::Matrix< Scalar, DimAtCompile, 1 > VectorT

**Definition:** bfgs.hpp:75

lbfgs\_update.hpp

stan::optimization::TERM\_LSFAIL

**Definition:** bfgs.hpp:29

stan::optimization::LSOptions

**Definition:** bfgs.hpp:56

stan::optimization::BFGSMinimizer::minimize

int minimize(VectorT &x0)

**Definition:** bfgs.hpp:276

stan::optimization::BFGSMinimizer::step

int step()

**Definition:** bfgs.hpp:158

stan::optimization::BFGSMinimizer::note

const std::string & note() const

**Definition:** bfgs.hpp:118

stan::optimization::BFGSMinimizer::get\_code\_string

std::string get\_code\_string(int retCode)

**Definition:** bfgs.hpp:120

stan::optimization::TERM\_RELF

**Definition:** bfgs.hpp:25

stan::optimization::TERM\_RELGRAD

**Definition:** bfgs.hpp:27

stan::optimization::BFGSLineSearch::grad

void grad(std::vector< double > &g)

**Definition:** bfgs.hpp:423

stan::optimization::LSOptions::minAlpha

Scalar minAlpha

**Definition:** bfgs.hpp:67

stan::optimization::ConvergenceOptions::fScale

Scalar fScale

**Definition:** bfgs.hpp:50

stan::optimization::BFGSMinimizer::prev\_p

const VectorT & prev\_p() const

**Definition:** bfgs.hpp:102

stan::optimization::BFGSMinimizer::initialize

void initialize(const VectorT &x0)

**Definition:** bfgs.hpp:145

stan::math::index\_type

Primary template class for the metaprogram to compute the index type of a container.

**Definition:** index\_type.hpp:21

stan::optimization::ModelAdaptor::df

int df(const Eigen::Matrix< double, Eigen::Dynamic, 1 > &x, Eigen::Matrix< double, Eigen::Dynamic, 1 > &g)

**Definition:** bfgs.hpp:385

stan::optimization::BFGSMinimizer::\_qn

QNUpdateType \_qn

**Definition:** bfgs.hpp:85

stan::optimization::BFGSLineSearch::grad\_evals

size\_t grad\_evals()

**Definition:** bfgs.hpp:420

stan::optimization::LSOptions::alpha0

Scalar alpha0

**Definition:** bfgs.hpp:66

util.hpp

stan::optimization::BFGSMinimizer::\_fk\_1

Scalar \_fk\_1

**Definition:** bfgs.hpp:81

stan::optimization::BFGSMinimizer::\_gk

VectorT \_gk

**Definition:** bfgs.hpp:80

stan::optimization::BFGSMinimizer::rel\_obj\_decrease

Scalar rel\_obj\_decrease() const

**Definition:** bfgs.hpp:108

stan::optimization::BFGSLineSearch::vector\_t

BFGSBase::VectorT vector\_t

**Definition:** bfgs.hpp:399

stan::optimization::BFGSLineSearch::logp

double logp()

**Definition:** bfgs.hpp:421

stan::optimization::TERM\_ABSGRAD

**Definition:** bfgs.hpp:26

stan::optimization::BFGSMinimizer::\_xk\_1

VectorT \_xk\_1

**Definition:** bfgs.hpp:80

stan::optimization::BFGSMinimizer::get\_qnupdate

const QNUpdateType & get\_qnupdate() const

**Definition:** bfgs.hpp:92

stan::optimization::BFGSMinimizer::prev\_step\_size

Scalar prev\_step\_size() const

**Definition:** bfgs.hpp:103

stan::optimization::BFGSMinimizer::\_gk\_1

VectorT \_gk\_1

**Definition:** bfgs.hpp:80

index\_type.hpp

stan::optimization::BFGSMinimizer::\_pk

VectorT \_pk

**Definition:** bfgs.hpp:80

stan::optimization::BFGSMinimizer::\_fk

Scalar \_fk

**Definition:** bfgs.hpp:81

stan::optimization::ModelAdaptor::operator()

int operator()(const Eigen::Matrix< double, Eigen::Dynamic, 1 > &x, double &f, Eigen::Matrix< double, Eigen::Dynamic, 1 > &g)

**Definition:** bfgs.hpp:341

stan::optimization::ModelAdaptor::fevals

size\_t fevals() const

**Definition:** bfgs.hpp:311

stan::optimization::BFGSMinimizer::\_func

FunctorType & \_func

**Definition:** bfgs.hpp:79

Eigen.hpp

stan::optimization::BFGSMinimizer::alpha

const Scalar & alpha() const

**Definition:** bfgs.hpp:115

stan::optimization::BFGSMinimizer::\_itNum

size\_t \_itNum

**Definition:** bfgs.hpp:83

bfgs\_update.hpp

stan::optimization::BFGSMinimizer::HessianT

Eigen::Matrix< Scalar, DimAtCompile, DimAtCompile > HessianT

**Definition:** bfgs.hpp:76

stan::optimization::CubicInterp

Scalar CubicInterp(const Scalar &df0, const Scalar &x1, const Scalar &f1, const Scalar &df1, const Scalar &loX, const Scalar &hiX)

Find the minima in an interval [loX, hiX] of a cubic function which interpolates the points...

**Definition:** bfgs\_linesearch.hpp:35

stan::optimization::ConvergenceOptions::ConvergenceOptions

ConvergenceOptions()

**Definition:** bfgs.hpp:35

stan::optimization::BFGSMinimizer::\_ls\_opts

LSOptions< Scalar > \_ls\_opts

**Definition:** bfgs.hpp:88

stan::math::e

double e()

Return the base of the natural logarithm.

**Definition:** constants.hpp:86

stan::optimization::BFGSMinimizer::prev\_x

const VectorT & prev\_x() const

**Definition:** bfgs.hpp:100

stan::optimization::BFGSLineSearch::BFGSLineSearch

BFGSLineSearch(M &model, const std::vector< double > &params\_r, const std::vector< int > &params\_i, std::ostream \*msgs=0)

**Definition:** bfgs.hpp:402

stan::optimization::BFGSMinimizer

**Definition:** bfgs.hpp:73

stan::optimization::BFGSMinimizer::curr\_p

const VectorT & curr\_p() const

**Definition:** bfgs.hpp:97

stan::optimization::BFGSMinimizer::curr\_f

const Scalar & curr\_f() const

**Definition:** bfgs.hpp:94

stan::math::min

double min(const double a, const double b)

**Definition:** min.hpp:7

stan::optimization::BFGSLineSearch::initialize

void initialize(const std::vector< double > &params\_r)

**Definition:** bfgs.hpp:412

stan::optimization::BFGSLineSearch

**Definition:** bfgs.hpp:394

stan::optimization::ModelAdaptor::operator()

int operator()(const Eigen::Matrix< double, Eigen::Dynamic, 1 > &x, double &f)

**Definition:** bfgs.hpp:312

bfgs\_linesearch.hpp

stan::optimization::BFGSMinimizer::rel\_grad\_norm

Scalar rel\_grad\_norm() const

**Definition:** bfgs.hpp:105

stan::optimization::ConvergenceOptions::tolRelGrad

Scalar tolRelGrad

**Definition:** bfgs.hpp:52

stan::optimization::BFGSMinimizer::curr\_g

const VectorT & curr\_g() const

**Definition:** bfgs.hpp:96

stan::optimization::LSOptions::LSOptions

LSOptions()

**Definition:** bfgs.hpp:58

stan::optimization::TERM\_ABSF

**Definition:** bfgs.hpp:24

stan::optimization::lp\_no\_jacobian

double lp\_no\_jacobian(const M &model, std::vector< double > &params\_r, std::vector< int > &params\_i, std::ostream \*o=0)

**Definition:** bfgs.hpp:288

stan::optimization::LSOptions::c2

Scalar c2

**Definition:** bfgs.hpp:65

stan::model::log\_prob\_propto

double log\_prob\_propto(const M &model, std::vector< double > &params\_r, std::vector< int > &params\_i, std::ostream \*msgs=0)

Helper function to calculate log probability for double scalars up to a proportion.

**Definition:** util.hpp:41

stan::optimization::ConvergenceOptions::tolAbsGrad

Scalar tolAbsGrad

**Definition:** bfgs.hpp:51

stan::optimization::ConvergenceOptions::tolAbsF

Scalar tolAbsF

**Definition:** bfgs.hpp:48

stan::optimization::BFGSMinimizer::\_alpha0

Scalar \_alpha0

**Definition:** bfgs.hpp:82

stan::optimization::ConvergenceOptions::tolRelF

Scalar tolRelF

**Definition:** bfgs.hpp:49


---

[ Stan Home Page ]

*© 2011–2014,
Stan Development Team.*
